# Immunopanning purification and long-term culture of human retinal ganglion cells

**Published:** 2010-12-28

**Authors:** Xin-Mei Zhang, David Ta Li Liu, Sylvia Wai-Yee Chiang, Kwong-Wai Choy, Chi-Pui Pang, Dennis Shun-Chiu Lam, Gary Hin-Fai Yam

**Affiliations:** 1 Department of Ophthalmology and Visual Sciences, The Chinese University of Hong Kong, Hong Kong, China; 2 Department of Obstetrics and Gynaecology, The Chinese University of Hong Kong, Hong Kong, China

## Abstract

**Purpose:**

To establish a robust method to isolate primary retinal ganglion cells (RGCs) from human fetal retina for long-term culture while maintaining neuronal morphology and marker protein expression.

**Methods:**

A total of six human retinas were obtained from aborted fetuses at 10 to 12 weeks of gestation with informed consent from mothers. RGCs were isolated and purified by a modified two-step immunopanning procedure. The cells were maintained in a serum-free defined medium supplemented with brain-derived neurotrophic factor, ciliary neutrophic factor, and forskolin. The viable RGCs and the extent of neurite outgrowth were examined by calcein-acetoxymethylester assay. Expression of RGC markers was studied by immunocytochemistry.

**Results:**

Primary RGCs from human fetal retinas were isolated and maintained in vitro for one month with substantial neurite elongation. In cell culture, almost 70% of the isolated cells attached, spread, and displayed numerous dendrites. They were immunoreactive to RGC-specific markers (Thy-1, TUJ-1, and Brn3a) and negative for glial fibrillary acidic protein and amacrine cells marker HPC-1.

**Conclusions:**

Human RGCs were successfully isolated and maintained in long-term culture. This can serve as an ideal model for biologic, toxicological, and genomic assays of human RGCs in vitro.

## Introduction

Retinal ganglion cells (RGCs) are the sole output neurons from the eyes, assuming the critical role of transmitting visual signals to the higher visual center at the brain cortex before signal processing [1]. They are the most important ocular cells; their anatomic or functional impairment is responsible for the development of most, if not all, ocular diseases and dysfunctions. The irreversible death of RGCs is associated with, or a consequence of, many ocular diseases, such as glaucoma and age-related macular degeneration (AMD), which are the leading causes of blindness worldwide [2]. Ocular neurodegeneration is rapidly becoming a global burden on the economy, social wellbeing, and the sustainability of health care systems. The mechanisms of RGC degeneration or death are complicated and current knowledge does not explain the disease-related RGC changes. How the morphological or functional changes of RGCs contribute to the development of diabetic retinopathy or glaucomatous optic neuropathy is largely unknown [3]. Accordingly, if the missing link between the cytopathological changes in RGCs and the development of glaucoma can be determined, studies about growth factors and RGC ion channel blockers may open up avenues for therapeutic neuroprotection [4]. Hence, dendritic growth, regeneration, and synapse formation are the most essential parameters for assessment of the cytoprotective or cytotoxic effects of various chemical molecules or exogenous pharmacological agents.

In view of the high research value of studying mammalian RGCs, there have been many studies on the early passages of mammalian RGCs in short-term explant culture [5-7]. Nevertheless, a mixed culture of different retinal cell types hinders a clear interpretation of the underlying cellular and molecular pathways attributed to RGCs. Moreover, poor cell viability due to cytokines released from dying cells in explants was a major drawback [8]. Enriched by a protocol reported in Barres et al. [9-11], RGCs and their culture were employed for studying intracellular signaling. The examination of neurite parameters, such as spanning area, neurite length, and branching, became technically feasible providing an objective, quantifiable, and reproducible measurement for studies on cell physiology and the pathophysiology of RGCs [8]. Transformed RGC lines may offer cells for studies on protein and RNA expressions but the lack of essential morphological and physiologic features of transformed RGCs, such as no dendritic processes or different patterns of electro-excitation and cytotoxicity, limit their usefulness [12].

There is a mounting need to develop a more versatile protocol for purification and long-term culture of primary human RGCs. In this study, we introduce a novel isolation technique using two-step immunopanning for harvesting human RGCs from fetal retinas and a sustained culture of RGCs with good preservation of neurite morphology and marker protein expression.

## Methods

### Human fetal retinal tissue

This research adhered to the tenets of the Declaration of Helsinki. With consent from the mothers, six fetal eyes were obtained from the donors, aborted at 10 to 12 weeks of gestation. These eyes were collected in sterile Neurobasal medium (Invitrogen, Carlsbad, CA) supplemented with 200 μg/ml penicillin G and 200 U/ml streptomycin sulfate (Invitrogen) on ice.

### Purification of retinal ganglion cells

The protocol was adapted from RGC purification of adult rat retina [9,10] with modifications. A two-step immunopanning procedure was performed.

#### Step 1. Dissociation of retina

Eye cups were dissected at the pars plana position, the retina was peeled from retinal pigment epithelium and trimmed to small pieces in Hank’s balanced salt solution (HBSS, Invitrogen) on ice. After gentle washing, the retinal pieces were incubated in an enzyme cocktail containing 70 U/ml crude collagenase (catalog no. C0130; Sigma, St Louis, MO), 15 U/ml papain (catalog no. P4762; Sigma), 0.2 mg/ml L-cysteine and 0.02% BSA (BSA; Sigma) for 10 min at 37 °C with gentle agitation. The cell suspension was triturated (vol:vol ratio of 1:5) with OVO-1 solution with 2 mg/ml Ovomucoid (catalog no. T2011; Sigma) and 1 mg/ml BSA in HBSS. It was further triturated (vol:vol ratio of 2:1) with OVO-2 solution with 10 mg/ml Ovomucoid and 10 mg/ml BSA in HBSS to yield a single cell suspension. After centrifugation, single cells were resuspended in HBSS containing 0.5 mg/ml BSA.

#### Step 2. Two-step immunopanning for RGCs

Sterile culture dishes (100 mm in diameter; Nunc, Roskilde, Rochester, NY) were prepared for immunopanning. They were coated with 10 µg/ml affinity-purified goat anti-mouse IgG (H^+^L) antibody (Jackson Immunology Lab, West Grove, PA) in 50 mM Tris (pH 9.5) at 4 °C overnight. After washing, the dish surface was blocked with 0.2 mg/ml BSA in 0.01 M PBS for 20 min. The dishes were then incubated with mouse anti-rat Thy1.1 antibody (2 µg/ml; catalog no. MAB1406; Chemicon, Temecula, CA) for 2 h at room temperature. After washing, the Thy1.1 antibody-coated dishes were stored at 4 °C and used within 2 days.

The retinal cell suspension was incubated with mouse anti-rat macrophage antiserum (anti-SIRP, catalog no. MAB1407P; Millipore, Billerica, MA) for 10 min and then centrifuged. The pellet was resuspended in 0.5 mg/ml BSA solution, and placed in a dish pre-coated with affinity-purified goat anti-mouse IgG (H^+^L) antibody for 45 min at room temperature. The plate was agitated every 15 min to ensure good cell adhesion. Non-adherent cells were transferred to the second dish pre-coated with goat anti-mouse IgG and incubated for another 30 min. The non-adherent cells were passed through a Nitex mesh filter (20 µm pore size; Wildco, Buffalo, NY) to obtain single cells, then put in the dish pre-coated with mouse anti-rat Thy1.1 antibody and incubated for 1 h with agitation every 20 min. After removal of non-adherent cells (collected for non-RGC testing), the dish was washed three times with PBS. Adherent cells were then detached by trypsinization using 0.05% trypsin (1:250; Invitrogen) with 0.53 mM EDTA.Na_2_ (Sigma) in PBS for 5 min at 37 °C. The cells were collected in Neurobasal medium with 1 mg/ml trypsin inhibitor (Sigma) and centrifuged to obtain a cell pellet.

After washing with PBS, the cells were resuspended in serum-free Neurobasal medium with 1% BSA, selenium (6.7 ng/ml; Sigma), transferrin (5.5 μg/ml; Sigma), putrescine (60 μM; Sigma), 3,5,3-triiodothyronine T3 (100 nM; Sigma), progesterone (20 nM; Sigma), B27 (Invitrogen), sodium pyruvate (1 mM; Invitrogen), glutamine (2 mM; Invitrogen), brain-derived neurotrophic factor (BDNF, 50 ng/ml; PeproTech, Rocky Hill, NJ), ciliary neurotrophic factor (CNTF, 10 ng/ml; PeproTech) and forskolin (5 μM; Sigma) and antibiotics added, and plated at a density of 5,000 cells/cm^2^ on a surface pre-coated with poly-L-lysine and laminin. The medium was replenished every 3 days.

### Cell viability

Isolated cells showing neurite outgrowth were viewed by adding 1 µM calcein-acetoxymethyl ester (calcein-AM; Molec Probes, Eugene, OR) in PBS for 60 min at 37 °C and then examining under fluorescence microscopy (excitation wavelength at 365 nm; emission wavelength at 430 nm; DMIRS; Leica, Wetzlar, Germany). The cells with typical neurite outgrowth were quantified and percentage of viability was obtained at different time points. The experiment was performed in triplicate.

### Immunocytochemistry

Cells were plated in Lab-Tek 8-chamber slides (EMS, Hatfield, PA) and fixed in freshly prepared 4% neutral buffered paraformaldehyde (Sigma) for 20 min. After PBS washes, they were permeabilized and blocked with PBS containing 0.1% Triton X-100 (Sigma) and 1% BSA for 10 min, followed by incubation with mouse monoclonal anti-human Thy-1 antibody (Santa Cruz Biotech., Santa Cruz, CA), mouse monoclonal anti-human βIII tubulin (Sigma), mouse monoclonal anti-human Brn3a (Chemicon), mouse monoclonal anti-human HPC-1 (Dako, Glostrup, Denmark), or rabbit polyclonal anti-human glial fibrillary acidic protein (GFAP; Dako) for 2 h at room temperature. After washing, the samples were incubated with Cy3-conjugated goat anti-mouse or anti-rabbit IgG (Jackson ImmunoRes Lab) for 1 h and then rinsed with PBS. The samples were mounted in ProLong Antifade solution (Molec Probes) and examined by confocal microscopy.

## Results

### Primary human fetal RGC

**Figure 1:**
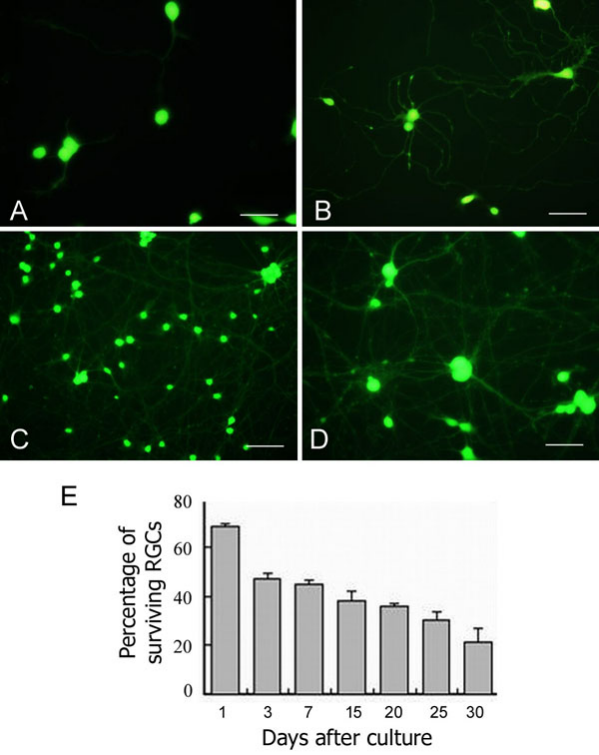
Isolated human retinal ganglion cells (RGCs) in culture. **A**-**D**: Morphological changes of human RGCs in serum-free defined culture at different time intervals. The cell bodies and neurites were examined by calcein-AM staining. (**A**) day 1; (**B**) day 7; (**C**) day 21 at lower magnification; (**D**) day 21 at higher magnification. **E**: Percentage of surviving RGCs over time. Approximately 50% of the RGCs survived after the first 3 days in culture. The survival percentages were moderately reduced and maintained at about 20% after 1 month. Experiments were performed in triplicate. Error bars: SD. Scale bars: (**A**, **B**, **D**) 50 µm, (**C**) 25 µm.

Human fetal retinal tissue was dissociated with collagenase and papain to single cells, which were subjected to a two-step immunopanning protocol to obtain an enriched population of RGCs. The cells were then propagated in a serum-free medium with supplements. At 24 h post-plating, more than 70% of the primary cells attached to the culture surface coated with laminin and poly-L-lysine. Through observation after calcein-AM staining, it was noted that most cells were oval in shape with little dendritic outgrowth (Figure 1A). At day 7, dendritic elongation from isolated RGCs was evident (Figure 1B) and similar observation was found in the majority of adherent cells. At day 21, most cells developed a complex dendritic network with the appearance of numerous neurites and branchings (Figure 1C,D). These primary human RGCs with extended neurites were maintained for up to one month in culture with the replenishment of fresh medium every 3 to 4 days. Quantification of calcein-AM-stained RGC cells showing neurites revealed that the viability declined with time (Figure 1E). Viability was approximately 50% at day 3 in culture and gradually reduced to about 25% at day 30, which was the longest time point in our examination.

### Characterization of primary human RGCs in culture

**Figure 2:**
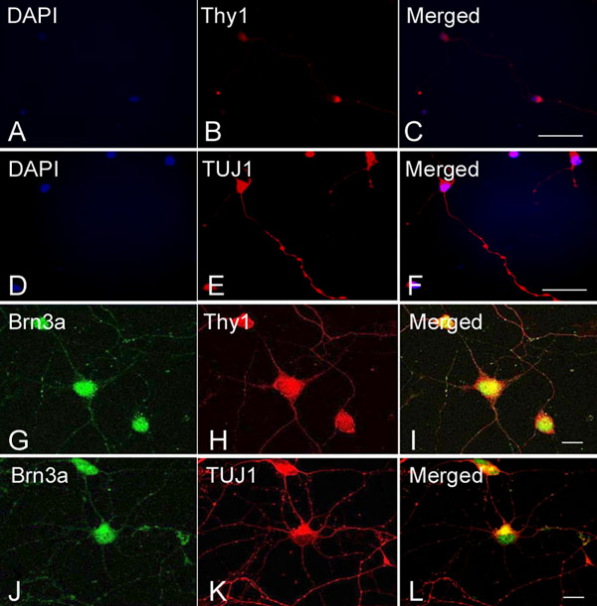
Expression of human retinal ganglion cell (RGC)-specific markers. **A**-**F**: Immunofluorescence of human RGCs at day 3 of culture shows positive expression of Thy-1 (**B**) and TUJ1 (**E**). DAPI nuclear staining is shown in (**A**) and (**D**) and in the merged images in (**C**) and (**F**). Confocal double immunofluorescence shows the co-staining of Brn3a (**G**) and Thy1 (**H**) in cells at day 3 of culture (merged image in **I**), and Brn3a (**J**) and TUJI (**K)** in cells at day 7 (merged image in **L**). Scale bars: 25 µm.

At days 3 and 7 of the first passage, primary RGCs were collected for immunocytochemistry of various RGC markers. Membranous staining of Thy1.1 was observed in the region of the soma and neurites (Figure 2B). DAPI nuclear staining to reveal RGCs is shown in Figure 2A and a merged image of Thy1.1 and DAPI is shown in Figure 2C. Immunoreactivity of TUJ1 was observed in the cytoplasm of the soma and neurites (Figure 2E) and compared to DAPI nuclear staining (Figure 2D) (merged picture in Figure 2F). By confocal microscopy, co-expression of Thy1.1 and Brn3a was observed in the RGCs at day 3. Thy1.1 was found on cell surfaces (Figure 2H), whereas Brn3a was found predominantly in the nuclei (Figure 2G) (merged picture in Figure 2I). At day 7, TUJ1 and Brn3a were detectable in RGCs (Figure 2J-L). To exclude the possibility of the presence of Muller or amacrine cells, we stained the cells for the expression of GFAP (marker for Muller cells) and HPC-1/syntaxin (marker for amacrine cells). The immunoreactivity of GFAP and HPC-1 was not detected in our cultured RGCs, but was confirmed positive in normal rat retinas (data not shown).

## Discussion

Cultured RGCs will be an important and almost indispensable tool for the study of retinal visual physiology and pathophysiology and it may easily evolve as state-of-art technology for studying the inter- or intra-cellular processes associated with various retinopathies and neuropathies [3]. Several cell culture models, including mixed retinal cells from neonatal or adult retinas [9,12], purified RGCs [10], transformed RGC cell lines [13], and retinal explant cells have been established in attempts to study retinal pathophysiology [14,15]. However, most studies had the shortcoming of species difference as the established cell cultures originated from rat or pig [16,17]. Therefore, there is an escalating need for the establishment of simple, reproducible, and reliable human RGC isolation and culture protocols with higher yield, better efficiency, good viability, and a longer period of RGC feature maintenance, for the sake of further research on retinal physiology and a better understanding of human retinal diseases.

Attempts to cultivate human RGCs with organ culture have been made but neurite outgrowth from explants was not observed [8]. When retina is mechanically dissected before culture, cells have poor viability. Supplementation of basic fibroblast growth factor allows cells to have better attachment and dendritic outgrowth with morphology similar to innate RGCs. However, problems with mixed growth of different retinal cell types and poor viability still persist. In the absence of a suitable in vitro RGC model, chemokine and toxicology studies on human RGCs had to be performed by immunohistochemistry methods in intact tissues, which presents its own pitfalls [18], such as antigenicity changes due to fixation and processing, epitope masking, and generation of non-specific backgrounds, from which result reproducibility and proper interpretation might be affected.

We have demonstrated a two-step immunopanning protocol to isolate human RGCs from fetal retinal tissues. The RGCs in culture showed dendritic outgrowth and elongation with axonal processes frequently contacting each other. They did not divide, and hence, the viability dropped over time, until about 25% at day 30 of culture. The cells were immunoreactive for RGC specific markers (Thy-1, TUJ-1, and Brn3a). Thy-1 is a surface glycoprotein of the immunoglobulin superfamily and has been shown to specifically express in RGCs [19]. The expression of Thy-1 in our cultured RGCs was similar to RGCs maintained in vivo [9]. TUJ1 (neuronal class III β-tubulin) is expressed in microtubules derived from rat brain and RGCs but not in retinal glial cells [20]. Brn3a is a class IV POU domain transcription factor responsible for the development of RGCs when ectopically expressed in retinal progenitor cells [21,22]. Our cultured cells were immuno-negative for Muller cell marker GFAP and amacrine cell marker HPC-1. In general, the expression pattern of these markers indicated that our culture method yielded a highly enriched RGC population.

This adherent culture of isolated human RGCs may allow the observation of growing dendrites. Quantitative parameters, such as neurite length, spanning area of neurites, and number and hierarchy of neurite branching, can be easily assessed by a vital staining protocol. Herein, we stained RGC bodies and neurites with calcein-AM, which became fluorescent when activated by an intracellular esterase in viable cells [23].

Continuous culture of RGCs or other adult CNS neurons is usually restricted by the post-mitotic characteristics of cells. In normal culture conditions, they do not divide, unless transformed with onco-genes or virus. Thus, the maintenance of cells with the preservation of initial phenotypes may be the most critical issue in RGC isolation. Our procedure was robust, since the isolated RGCs were maintained in “long-term” culture (which is defined as more than one week in most literature [4]) with expression of a panel of RGC-specific phenotypes. Because the RGC yield is greatly affected by lengthy post-mortem procedures and tissue handling, our study recruited human fetal eyes obtained 2 to 4 h after abortion. We dissociated the retinal tissues with an enzyme cocktail (70 U/ml crude collagenase, 15 U/ml papain in the presence of 0.2 mg/ml L-cysteine) for 10 min to single cells. This was 20 to 35 min shorter than previous reports isolating rat RGCs [10,24] and 50 min shorter than one for human RGCs [8]. This could account for the better cell survival and the capacity for neurite outgrowth. From enzymatic digestion of a single fetal retina, we obtained approximately 6×10^7^ retinal cells of mixed types. This was enriched to about 5×10^5^ RGCs after the two-step immunopanning isolation procedure. Moreover, we found no difference in the isolation efficiency of human RGCs with the use of anti-human Thy-1 (Chemicon) or anti-rat Thy-1.1 (Chemicon). For cost efficiency and result consistency, we recommend using anti-rat Thy-1.1 for RGC enrichment.

Our isolation protocol was developed and modified based on that used for the isolation of rat RGCs [10,24]. We prepared sterile culture dishes for immunopanning by first coating with an affinity-purified IgG (H^+^L) antibody, followed by another coating with anti-SIRP and anti-rat Thy-1.1. This contributed to more efficient trapping and a higher yield of RGC-like cells. We also prolonged the incubation time in the panning steps from 30 min to 1 h. In our serum-free defined culture with the common RGC survival factors (such as BDNF, CNTF, B27, and forskolin) in Neurobasal medium, we supplemented with selenium, putrescine, thryoxine, triiodothyronine, transferrin, and progesterone (collectively known as Botterstein-Sato formula) [25]. This resulted in a higher yield of enriched RGCs and promoted their survival and long-term maintenance in culture.

The successful isolation and culture of RGCs with high yield and efficiency provided an important laboratory protocol for the study of the effect of various biochemical or pharmacological factors on these important visual pathway cells, undoubtedly enhancing our understanding of the pathophysiology of blindness and common eye diseases, such as glaucoma or age-related macular degeneration. This novel RGC culture procedure could prove valuable in future molecular, cellular, physiologic, pathophysiological, and pharmacological studies, and in almost every other branch of basic and clinical research pertaining to RGC-related ocular diseases.
